# The association between physical activity, fitness and body mass index on mental well-being and quality of life in adolescents

**DOI:** 10.1007/s11136-018-1915-3

**Published:** 2018-06-12

**Authors:** William T. B. Eddolls, Melitta A. McNarry, Leanne Lester, Charles O. N. Winn, Gareth Stratton, Kelly A. Mackintosh

**Affiliations:** 1 Applied Sports Technology, Exercise and Medicine (A-STEM) Research Centre, College of Engineering, Bay Campus, Swansea University, Swansea, UK; 2 School of Human Sciences, University of Western Australia, Perth, Australia; 3 Swansea University Medical School, Singleton Campus, Swansea University, Swansea, UK; 4 School of Sport Health and Exercise Science, University of Western Australia, Perth, Australia

**Keywords:** Physical activity, Cardiorespiratory fitness, Body mass index, Mental well-being, Quality of life, Adolescents

## Abstract

**Purpose:**

The purpose of the current study was to investigate the mediatory role between vigorous physical activity, body mass index (BMI), and cardiorespiratory fitness on symptoms of depression and their subsequent direct and indirect effects on quality of life (QoL).

**Methods:**

Five hundred and seventy-six adolescents’ (314 boys, 12.5 ± 1.1 years) physical activity levels, cardiorespiratory fitness, BMI, levels of depressive symptoms, and QoL were measured. Structural equation modelling was used to evaluate the difference in linear structural associations between variables.

**Results:**

The model suggested that cardiorespiratory fitness (*β* = 0.16, *p* < 0.001) and symptoms of depression (*β* = − 0.52, *p* < 0.001) were both directly associated with physical QoL, with depressive symptoms also directly influencing psychological QoL (*β* = − 0.79, *p* < 0.01). Body mass index was indirectly associated with physical QoL, mediated by both symptoms of depression (*β* = − 0.06, *p* < 0.001) and cardiorespiratory fitness (*β* = 0.05, *p* < 0.001) and psychological QoL mediated by symptoms of depression (*β* = − 0.09, *p* < 0.001). Vigorous physical activity was indirectly associated with QoL, mediated by cardiorespiratory fitness (*β* = − 0.04, *p* < 0.001).

**Conclusions:**

Models suggested that vigorous physical activity, cardiorespiratory fitness and BMI were associated, both directly and indirectly, with mental well-being and QoL. It could, therefore, be postulated that enhancing cardiorespiratory fitness and BMI through increasing vigorous physical activity may be beneficial to both mental well-being and QoL in adolescents.

## Introduction

Quality of life (QoL), a subset of health defined by the World Health Organisation to include the physical, mental and social well-being of a person [1], is widely recognised as a fundamental element in the evaluation of population health [2] and should be considered the underlying target for health interventions [3]. Nonetheless, despite its widely accepted importance, recent findings have suggested that self-reported QoL has significantly declined in adolescents over the last decade [4], highlighting the need for interventions that specifically target QoL in this population. However, due to the subjective nature of QoL, it cannot be directly enhanced [5]. Therefore, the influence of health-related parameters warrants further attention as potential mediators through which to enhance QoL.

Previous research has shown that interventions that modify certain health-related parameters associated with QoL, such as physical health and mental well-being, can indirectly elicit improvements in QoL. Cardiorespiratory fitness and body mass index (BMI), inter-related indicators of health [6], predict QoL; those with a higher cardiorespiratory fitness experience a better QoL [7], and overweight or obese individuals typically experiencing a poorer QoL [8]. Cardiorespiratory fitness and BMI have also been associated with additional health-related parameters that can also influence QoL, namely, mental well-being and physical activity [9, 10]. Indeed, research has suggested that overweight or obese individuals and those with low cardiorespiratory fitness are less physically active and often have poorer mental health. Specifically relating to QoL, those suffering from mental health issues, or not meeting physical activity guidelines of 60 min of moderate-to-vigorous physical activity per day, are at greater risk of a poor QoL [11, 12]. However, despite these predictors being identified, the interaction between them and the influence of mental health on QoL remains to be elucidated. Indeed, little research has explored the potential indirect relationships between health behaviours and their mediating and interactive influence on QoL. For example, when compared to moderate physical activity, vigorous physical activity has been associated with fewer symptoms of depression [13], the most common form of mental illness in the UK [14], and has also been shown to predict cardiorespiratory fitness and adiposity [10]. Thus, vigorous physical activity levels have the potential to indirectly affect QoL through symptoms of depression, adiposity and cardiovascular fitness.

Therefore, the purpose of the current study was to investigate the relationships between vigorous physical activity, BMI and cardiorespiratory fitness on symptoms of depression, and their subsequent direct and indirect effects on QoL using structural equation modelling.

## Methods

### Participants

Five hundred and seventy-six adolescents (314 boys, 12.5 ± 1.1 years) took part in the study. Following parental consent and child assent, measures were taken over approximately 6 weeks at the beginning of the academic year (September–October). All participants were from multiple, randomly stratified, comprehensive schools located in South Wales, UK. Approval to conduct this research was granted by the institutional ethical advisory committee (ethics number: PG/2014/29) and performed in accordance with the Declaration of Helsinki.

### Measures

#### Anthropometric and maturity assessment

Stature and body mass were measured, to the nearest 0.1 cm and 0.1 kg, respectively, using a stadiometer (Seca 213, Seca Ltd, Birmingham, UK) and body mass scales (Seca 899, Seca Ltd, Birmingham, UK). From these measures, BMI was calculated. Maturational status was assessed using self-reported indices of pubic hair, as described by Tanner [15].

#### Physical activity levels

The GT3X+ (ActiGraph, Pensacola, FL, USA) was used to measure and record the quantity and frequency of body movement, providing a valid and reliable objective measure of physical activity [16]. Participants were asked to wear the accelerometer, set at 100 Hz, on their right mid-axilla line at the level of the iliac crest for seven full days, only removing it if they undertook contact or water-based activities. Wear-time diaries were used to log why the accelerometers were taken off and for how long. Data were analysed using KineSoft (version 3.3.67; KineSoft, Saskatchewan, Canada) employing 1 s epochs with sustained periods of at least 20 min at zero counts considered non-wear time [17]. A minimum daily wear time of 10 h for 3 days, including one weekend day, was set, above a recommended criterion [18]. Everson et al. [19] cut points, shown to be valid and reliable determinants of activity intensity in children and adolescents [20], were used to calculate vigorous physical activity (> 4012 counts per minute).

#### Depressive symptoms measurement

Symptoms of depression were measured using the Center for Epidemiologic Studies Depression Scale for Children (CES-DC) questionnaire [21], which has been validated for use within the adolescent population [22]. The CES-DC is a 20-item scale with overall scores ranging from 0 to 60 with higher scores representing a greater level of depressive symptoms. Internal reliability for this scale, based on Cronbach’s Alpha [23], was deemed good (*α* = 0.91).

#### Quality of life measurement

Perceived QoL was assessed using the Pediatric Quality of Life Inventory (PedsQL) Teenager Report, Version 4.0 [24], a widely validated 23-item scale designed for use with participants aged between 13 and 18 years [25]. Within the construct of the 23-item questionnaire, the measure is broken down into four scales: physical (*n* = 8), emotional (*n* = 5), social (*n* = 5) and school functioning (*n* = 5). Physical (*n* = 8) and psychological (*n* = 15) QoL summary scores were calculated. Scores ranged between 0 and 100, with higher scores indicative of a better QoL. Internal reliabilities for physical QoL (*α* = 0.759) and psychological QoL (*α* = 0.875) were deemed acceptable [23].

#### Cardiorespiratory fitness

Cardiorespiratory fitness was estimated using the multi-stage fitness test, a previously validated field measure in children [26]. The multi-stage fitness test was conducted over a 20-m space, in a hard-floor indoor sports hall, with the total number of shuttles completed used as a score marker.

### Statistical analysis

Analysis was guided by Ferrans et al. [27] conceptual model, which hypothesised that measured health outcomes can predict QoL. Data were analysed using IBM SPSS Statistics for Windows, Version 22.0 (IBM Corp, Armonk, NY, 2013) and IBM SPSS AMOS for Windows, Version 22.0 (IBM Corp, Armonk, NY, 2013). Missing data (*n* = 903, 17.4%) were imputed using an expectation–maximisation algorithm. Structural equation modelling was used to evaluate the difference in linear structural associations between variables. Direct effects were estimated using direct path coefficients between two measured variables and indirect associations were estimated as a product of two direct effects between three measured variables. Given the potential confounding effects in children and adolescents, sex and maturation were accounted for within the model. All data are presented as means ± standard deviation (SD) with a statistically significant criterion set at *p* < 0.05. For analysis, unless otherwise stated, data were standardised (β).

## Results

The means and SD of the measured variables of the children and adolescents that participated in this study, dichotomised by sex, are presented in Table 1.

**Table 1:** Means and SD of the measured variables of the study participants, dichotomised by sex with squared multiple correlation coefficients (*R*^2^)

	Boys (*n* = 314)	Girls (*n* = 262)	*R* ^2^
BMI	20.93 ± 4.11	20.69 ± 3.74	.104
MSFT	46.9 ± 23.1	33.2 ± 14.7	.368
CES-DC	14.3 ± 10.0	20.3 ± 11.6	.088
PedsQL (physical)	81.09 ± 14.86	77.14 ± 14.83	.318
PedsQL (psychological)	78.58 ± 15.79	73.30 ± 16.97	.620
VPA	26.09 ± 10.21	22.75 ± 7.64	.032

*SD* standard deviation, *MSFT* multi-stage fitness test, *BMI* body mass index, *CES-DC* Center for Epidemiologic Studies Depression Scale, *PedsQL* Pediatric quality of life inventory, *VPA* vigorous physical activity

### Confirmatory factor analysis

**Fig. 1:**
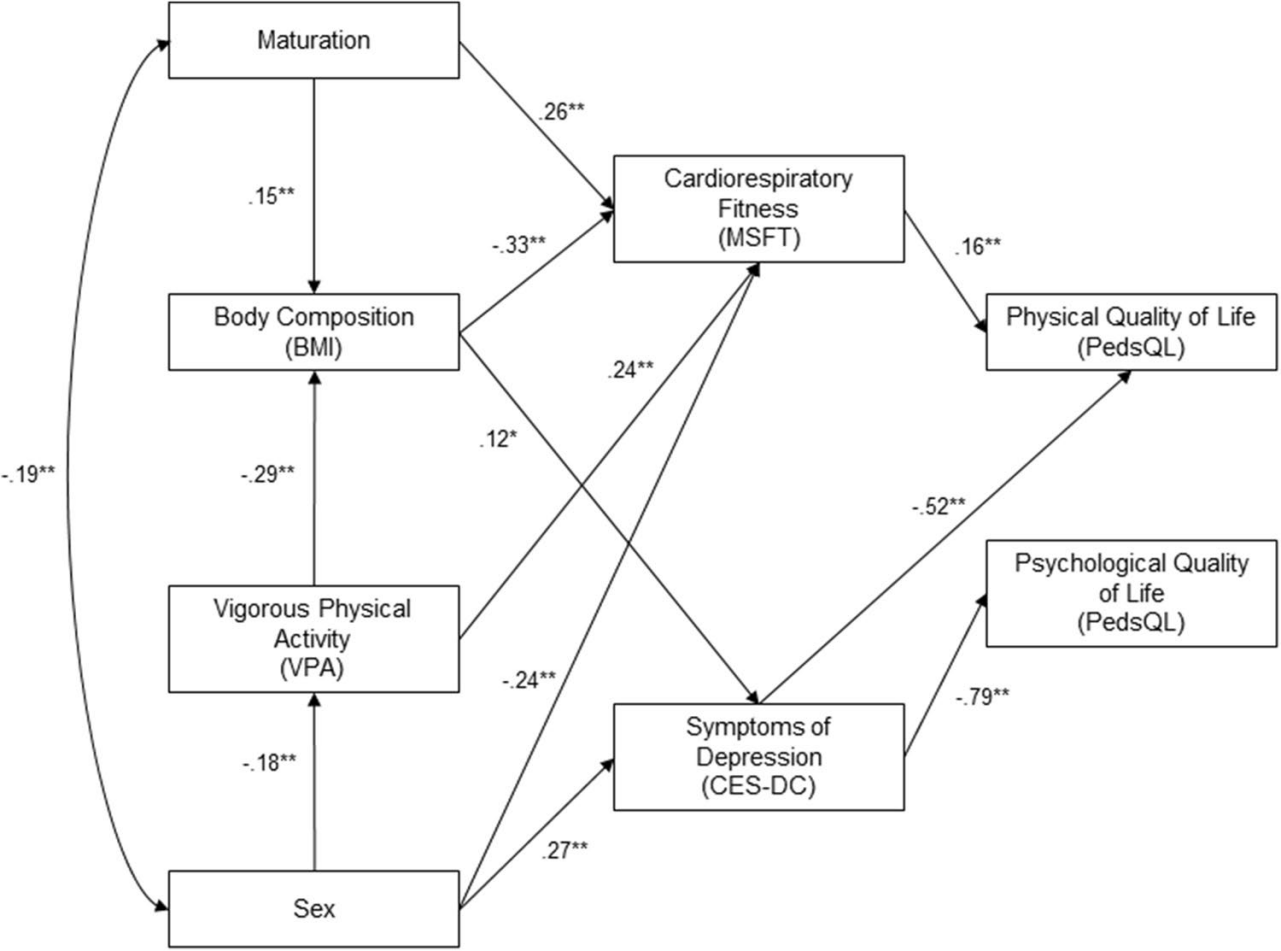
Structural equation model of physical activity levels, physiological health and their effect on mental well-being and quality of life in adolescents with standardised beta coefficients (*p < 0.05, **p < 0.01)

Inspection of the model fit indices (NFI = 0.981, TLI = 0.982, CFI = 0.991, RMSEA < 0.05) suggested that the model was acceptable. Standardised parameter estimates for the measurement model are provided in Fig. 1.

### Structural equation model

Standardised and unstandardised regression weights with standard errors between variables included within the model are provided within Table 2.

**Table 2:** Standardised and unstandardised regression weights with standard errors

Paths	β	B	SE
Sex → VPA**	− 0.18	− 3.34	0.76
Maturation → BMI**	0.15	0.55	0.15
VPA → BMI**	− 0.29	− 0.13	0.02
Maturation → MSFT**	0.26	0.53	0.07
BMI → MSFT**	− 0.33	− 0.18	0.02
BMI → CES-DC*	0.12	0.33	0.11
VPA → MSFT**	0.24	0.06	0.01
Sex → CES-DC**	0.27	6.04	0.89
Sex → MSFT**	− 0.24	− 1.04	0.15
CES-DC → PedsQL (psychological)**	− 0.79	− 1.17	0.04
MSFT → PedsQL (physical)**	0.16	0.89	0.20
CES-DC → PedsQL (physical)**	− 0.52	− 0.70	0.05

*β* standardised regression weight, *B* unstandardised regression weight, *SE* standard error, *MSFT* multi-stage fitness test, *BMI* body mass index, *CES-DC* Center for Epidemiologic Studies Depression Scale, *PedsQL* pediatric quality of life inventory, *VPA* vigorous physical activity

*p < 0.05, **p < 0.01

### Vigorous physical activity

The model (Fig. 1) revealed that vigorous physical activity was directly related to BMI (*β* = − 0.29, *p* < 0.001) and cardiorespiratory fitness (*β* = 0.24, *p* < 0.05), and indirectly related to depressive symptoms scores (*β* = − 0.03, *p* < 0.001) and physical QoL (*β* = − 0.04, *p* < 0.001), mediated by BMI and cardiorespiratory fitness, respectively.

### Body mass index

Body mass index was directly related to cardiorespiratory fitness (*β* = − 0.33, *p* < 0.001) and depressive symptoms scores (*β* = 0.12, *p* < 0.05). Indirect relationships were found between BMI and physical QoL, mediated by cardiorespiratory fitness (*β* = 0.05, *p* < 0.001) and symptoms of depression (*β* = − 0.06, *p* < 0.001). Additionally, an indirect relationship was found between BMI and psychological QoL (*β* = − 0.09, *p* < 0.001), mediated by symptoms of depression.

### Cardiorespiratory fitness

Cardiorespiratory fitness had direct and indirect effects on QoL measures; cardiorespiratory fitness scores had a direct relationship with physical QoL (*β* = 0.16, *p* < 0.001), and an indirect relationship with overall QoL (*β* = 0.06, *p* < 0.001), mediated by physical QoL.

### Depression

There was a direct relationship between symptoms of depression and psychological QoL (*β* = − 0.79, *p* < 0.01) and physical QoL (*β* = − 0.52, *p* < 0.001) and an indirect relationship with overall QoL mediated by physical QoL (*β* = − 0.20, *p* < 0.001) and psychological QoL (*β* = − 0.61, *p* < 0.001).

### Maturation and sex

Maturation directly affected BMI (*β* = 0.15, *p* < 0.001) and cardiorespiratory fitness scores (*β* = 0.26, *p* < 0.001), whilst sex was found to directly affect vigorous physical activity (*β* = − 0.18, *p* < 0.01), cardiorespiratory fitness (*β* = − 0.24, *p* < 0.001) and depressive symptoms (*β* = 0.27, *p* < 0.001).

## Discussion

This study investigated the relationship and mediatory effects between physical activity levels, BMI, cardiorespiratory fitness and symptoms of depression, and their subsequent effect on physical and psychological QoL. Models suggested that vigorous physical activity, cardiorespiratory fitness and BMI were associated, both directly and indirectly, with mental well-being and physical and psychological QoL. It could, therefore, be postulated that enhancing cardiorespiratory fitness and BMI through increasing vigorous physical activity may be beneficial to both mental well-being and QoL in adolescents.

The current results show that, in accord with previous literature [28], a healthier BMI is associated with fewer symptoms of depression. It is possible that this association, at least in part, may be due to psychosocial factors associated with BMI. For example, stigmatisation towards those that have a higher BMI may cause lower self-esteem, induce negative self-images and lead to perceptions of social isolation, thus eliciting depressive symptoms [29]. A review by Markowitz et al. [30] also proposes that the association between obesity and depression is related to concerns regarding self-perceived health and appearance, whereby those with higher BMI experience, or are more vulnerable to, depression.

As alluded to previously, symptoms of depression were also associated with physical and psychological QoL. Indeed, congruent with previous research [11], findings from the present study suggest that lower levels of depressive symptoms were associated with higher levels of psychological and physical QoL, highlighting the link between mental well-being and QoL. In addition to the direct associations with depressive symptoms, the model also proposes that a healthier BMI is indirectly associated with a greater psychological and physical QoL, mediated by levels of depressive symptoms and cardiorespiratory fitness, respectively. As outlined in previous literature [31], these findings, therefore, identify BMI as a key influential factor that can significantly affect an individual’s QoL. Indeed, interventions designed to improve QoL via a reduction in BMI have reported positive results [32].

Beyond depressive symptoms and BMI, participants with a higher cardiorespiratory fitness also experienced a better physical QoL [7]. Given the link between cardiorespiratory fitness and BMI observed in this study and elsewhere [33], the association between cardiorespiratory fitness and physical QoL [34] may also be related to a negative self-image and self-esteem [29], components that are encompassed within the QoL measurement. In addition to psychosocial issues, given that disease impairs QoL [35], it could be postulated that the effect of cardiorespiratory fitness on physical QoL may be related to its association with an increased risk of physical morbidities [36]. However, the physiological mechanisms explaining the connection between cardiorespiratory fitness and QoL are not entirely understood [37] and, therefore, warrant further research. Subsequently, future interventions seeking to enhance QoL should remain multi-faceted, simultaneously improving cardiorespiratory fitness, BMI and symptoms of depression.

A further interesting finding was that, whilst higher levels of vigorous physical activity were directly associated with lower BMI and increased cardiorespiratory fitness [38, 39], higher levels of vigorous physical activity were also indirectly associated with fewer symptoms of depression, mediated by BMI. It is postulated that, the negative correlation between vigorous physical activity and BMI may be associated with improvements in symptoms of depression, potentially due to greater regulation of the hypothalamic–pituitary–adrenal axis and insulin control, as well as improved self-perceptions in both appearance and health [29, 30, 40]. Consequently, increasing vigorous physical activity may elicit improvements to not only a person’s physical health, but also may improve mental well-being. Indeed, Fox [41] found that healthy adults should consider vigorous exercise as a means of improving mental well-being. However, with the exception of Parfitt et al. [42] and Gerber et al. [13], who found vigorous physical activity elicited mental health benefits beyond those engendered through low and moderate physical activity, there is limited research that focuses on the effects of vigorous physical activity on mental well-being, especially in children and adolescents. Given these findings [13, 42], in addition to the identification of vigorous physical activity as a moderator of mental health within the current study, whilst exercise interventions have focused on high volume, moderate-intensity or resistance-based exercise [43], alternative forms or intensities of exercise may be more beneficial. Subsequently, despite previous mixed findings [44, 45], the current study presents a newfound advocacy for the potential of high-intensity exercise, a structured form of vigorous physical activity, to directly and indirectly improve symptoms of depression and physical and psychological QoL, mediated through the well-documented physiological health benefits [46].

Congruent with previous research [47], the present study demonstrated that sex had a significant relationship with both cardiorespiratory fitness and symptoms of depression; girls were more likely to experience poorer fitness and mental well-being. These differences may, in part, be related to environmental considerations, for example, sociocultural roles and psychological attributes [48] or hormonal factors [49]. However, it is also important to consider the importance of the divergence in physical development during puberty, such as changes to lean muscle and fat [47] relating to psychosocial factors associated with overweight or obesity [29, 30], therefore, further highlighting the need for health interventions to primarily focus on improving BMI to enhance overall health.

This is the first study to use structural equation modelling to determine both direct and indirect associations between physiological health, physical activity levels, mental well-being and QoL. Furthermore, the present study accounted for maturational status and sex, both of which are often overlooked in child and adolescent studies, as well as using robust, previously validated, measures of physical activity [16]. Nonetheless, whilst this study is associated with numerous strengths, there are inevitably some limitations. It is anticipated that the omission of socio-economic status within the model, given its well-established relationship with the measured variables [50], may limit the interpretation of the findings. Despite its frequent utilisation within clinical research, the application of BMI is commonly subject to scepticism due to the measure’s inability to differentiate between lean and fat body mass [51]. Additional limitations include the self-reporting of mental well-being and the associated disadvantages, such as social desirability [52]. Furthermore, for the QoL measurement a number of participants fell below the minimum age threshold for the Teenager Report (13–18 tears), however, given that there are only small differences between Child Report and Teenage Report (i.e., replacing the word child with teenager), although it cannot be ruled out, it was deemed unlikely to have influenced the results. However, all of the questionnaires used were previously validated [22, 25] and demonstrated acceptable reliability. Finally, primarily due to participants failing to meet accelerometer wear-time criteria, expectation–maximisation was utilised to impute missing data, and whilst it has previously been reported as a valid method [53], it is important to acknowledge the influence the absence of a complete data set may have had on the findings within this study.

## Conclusions

In conclusion, the present study found that vigorous physical activity, cardiorespiratory fitness and BMI were associated, both directly and indirectly, with mental well-being and physical and psychological QoL. As such, it could be suggested that improving cardiorespiratory fitness and BMI by increasing vigorous physical activity may be beneficial to both mental well-being and QoL in adolescents. Subsequently, our findings suggest that, to elicit improvements to QoL, exercise or physical activity interventions should remain multi-faceted, simultaneously focusing on cardiorespiratory fitness, BMI and symptoms of depression.
